# Isotropic thermal insulating cuttlebone-inspired MXene aerogel

**DOI:** 10.1093/nsr/nwaf342

**Published:** 2025-08-25

**Authors:** Junsong Fu, Wangwei Lian, Yankang Deng, Zixuan Fang, Qunfeng Cheng

**Affiliations:** State Key Laboratory of Bioinspired Interfacial Materials Science, School of Nano Science and Technology, Suzhou Institute for Advanced Research, University of Science and Technology of China, Suzhou 215123, China; State Key Laboratory of Bioinspired Interfacial Materials Science, School of Chemistry, Key Laboratory of Bio-inspired Smart Interfacial Science and Technology of Ministry of Education, Beihang University, Beijing 100191, China; State Key Laboratory of Bioinspired Interfacial Materials Science, School of Chemistry and Materials Science, University of Science and Technology of China, Hefei 230026, China; State Key Laboratory of Bioinspired Interfacial Materials Science, School of Nano Science and Technology, Suzhou Institute for Advanced Research, University of Science and Technology of China, Suzhou 215123, China; State Key Laboratory of Bioinspired Interfacial Materials Science, School of Chemistry, Key Laboratory of Bio-inspired Smart Interfacial Science and Technology of Ministry of Education, Beihang University, Beijing 100191, China; State Key Laboratory of Bioinspired Interfacial Materials Science, School of Chemistry and Materials Science, University of Science and Technology of China, Hefei 230026, China; State Key Laboratory of Bioinspired Interfacial Materials Science, School of Nano Science and Technology, Suzhou Institute for Advanced Research, University of Science and Technology of China, Suzhou 215123, China; State Key Laboratory of Bioinspired Interfacial Materials Science, School of Chemistry, Key Laboratory of Bio-inspired Smart Interfacial Science and Technology of Ministry of Education, Beihang University, Beijing 100191, China; State Key Laboratory of Bioinspired Interfacial Materials Science, School of Chemistry and Materials Science, University of Science and Technology of China, Hefei 230026, China; State Key Laboratory of Bioinspired Interfacial Materials Science, School of Nano Science and Technology, Suzhou Institute for Advanced Research, University of Science and Technology of China, Suzhou 215123, China; State Key Laboratory of Bioinspired Interfacial Materials Science, School of Chemistry, Key Laboratory of Bio-inspired Smart Interfacial Science and Technology of Ministry of Education, Beihang University, Beijing 100191, China; State Key Laboratory of Bioinspired Interfacial Materials Science, School of Chemistry and Materials Science, University of Science and Technology of China, Hefei 230026, China; State Key Laboratory of Bioinspired Interfacial Materials Science, School of Nano Science and Technology, Suzhou Institute for Advanced Research, University of Science and Technology of China, Suzhou 215123, China; State Key Laboratory of Bioinspired Interfacial Materials Science, School of Chemistry, Key Laboratory of Bio-inspired Smart Interfacial Science and Technology of Ministry of Education, Beihang University, Beijing 100191, China; State Key Laboratory of Bioinspired Interfacial Materials Science, School of Chemistry and Materials Science, University of Science and Technology of China, Hefei 230026, China; Institute of Energy Materials Science (IEMS), University of Shanghai for Science and Technology, Shanghai 200093, China

**Keywords:** MXene, aerogel, cuttlebone-bioinspired, mechanical properties, thermal insulating

## Abstract

Aerogels are considered to be ideal thermal insulation materials due to their low thermal conductivity and highly porous structure, which can effectively reduce the energy consumption in aerospace, industry, and building applications. However, the anisotropic performance of most aerogels results in high thermal conductivity in the axial direction, and structures tend to collapse under the extreme temperature shock, leading to poor mechanical stability. Herein, we demonstrate a remarkable lightweight and isotropic thermal insulation aerogel material inspired by the wall–septa microstructure of cuttlebone. The cuttlebone-inspired MXene aerogel (CMA) is fabricated through freeze casting colloidal suspensions composed of Ti_3_C_2_T*_x_* MXene nanosheets, montmorillonite nanosheets, cellulose nanofibers, and polyvinyl alcohol. The CMA shows an ultralow thermal conductivity of 17.1 mW m^−1^ K^−1^ in the radial direction and 19.7 mW m^−1^ K^−1^ in the axial direction. Additionally, the CMA also exhibits a rapid sensing response, robust fire resistance/fire warning, and excellent electromagnetic interference (EMI) shielding of ∼61 dB in both radial and axial directions. The structural integrity and EMI shielding performance remain stable over a wide temperature range (−196°C to 1300°C). This performance indicates the potential of CMA as a promising alternative to existing thermal insulation under extreme conditions.

## Teaser

A cuttlebone-inspired MXene aerogel with hierarchical porous structure, shows excellent thermal insulation in both radial and axial directions, rapid sensing response, robust fire resistance, and high electromagnetic interference shielding.

## INTRODUCTION

Since the beginning of the 21st century, issues such as energy crisis, global warming, and environmental pollution have become increasingly severe. Among these, carbon dioxide emissions are the primary driver of global warming, with building energy consumption accounting for >40% of total energy use and carbon dioxide emissions [1,2]. High-performance thermal insulation materials can mitigate the impact of external environmental conditions on indoor temperatures through their low thermal conductivity, thereby reducing the reliance on heating systems and achieving carbon emission reductions while maintaining indoor comfort [3,4]. Aerogels are regarded as ideal thermal insulation materials thanks to their high porosity and exceptionally low thermal conductivity [5,6]. The thermal insulation mechanism of aerogels lies in their pore size being smaller than the mean free path of air molecules at atmospheric pressure, rendering the air molecules within the pores nearly stationary and thus suppressing convective heat transfer. At the same time, the extremely low bulk density and tortuous pathways of the aerogel's porous structure inhibit heat conduction between the gas and the solid phase, while the near-‘infinite’ void walls minimize heat radiation [7,8].

As a low-cost, scalable, and environmentally friendly technology, freeze casting can effectively and precisely control the porous structure of materials [9–11]. This technique utilizes the directional growth of ice crystals to exclude the nanomaterials in the slurry, resulting in oriented porous aerogel structures after freeze-drying. Examples include ceramic nanofiber aerogels [12,13], carbon nanofiber aerogels [14], biomass-derived aerogels [15], boron nitride aerogels [16], and graphene aerogels [17]. Although these aerogels exhibit low thermal conductivity in the radial direction, their layered or honeycomb-like anisotropic structure led to ultrahigh thermal conductivity in the axial direction. More critically, these aerogels are prone to decomposition and pulverization under the high temperatures shock (>500°C) [18–21]. Furthermore, functional limitations hinder their further application in aerospace vehicles, as they are unable to provide adequate thermal and personal protection under extreme conditions [22,23].

Ti_3_C_2_T*_x_* MXene is an emerging class of two-dimensional (2D) nanomaterials with exceptional mechanical properties and high electrical conductivity [24–26]. MXene nanosheets have been regarded as ideal candidates for assembling high-performance macroscopic aerogels, driven by their promising applications in sensor, electromagnetic interference (EMI) shielding, and thermal management [27–30]. In addition, cuttlebone, a lightweight, high-strength porous biomineralizing material, can withstand pressures of up to 20 atmospheres in the deep sea [31,32]. This remarkable mechanical performance is attributed to its evolutionarily optimized ripple gradient wall–septa microstructure (Figs S1 and S2). The cuttlebone exhibits excellent compressive properties in both radial and axial directions, achieving isotropic damage tolerance and energy absorption (Fig. S3). Inspired by the cuttlebone's rigid cavity-wall structure, the excellent mechanical and energy absorption properties were achieved by layer-by-layer assembly of two types of predesigned hydrogels featuring both brick-and-mortar structures and close-packed rigid micro hollow structures [33]. Versatile cement aerogels were developed via freeze casting, demonstrating high strength, flame retardancy, and negative Poissonʼs ratio [34]. In addition, a biodegradable lignocellulosic foam was fabricated through a multiscale supramolecular assembly strategy, exhibiting light weight yet rigid characteristics while possessing excellent thermal insulation properties [35]. However, current cuttlebone-inspired aerogel or foam materials remain predominantly focused on mechanical properties, with insufficient research on thermal insulation and electromagnetic shielding performance. This limitation hinders their further application in extreme environments such as the aerospace and building fields [36]. Therefore, it is imperative to develop lightweight and high-strength aerogels with isotropic thermal insulation properties.

Drawing inspiration from the hierarchical wall–septa microstructure of cuttlebone, we demonstrated a novel cuttlebone-inspired MXene aerogel (CMA) with exceptional isotropic thermal insulation properties, fabricated through bidirectional freeze casting. The CMA demonstrates an ultralow thermal conductivity of 17.1 mW m^−1^ K^−1^ in the radial direction and 19.7 mW m^−1^ K^−1^ in the axial direction. Moreover, the CMA also shows rapid sensing response, robust fire resistance, smart fire warning, and high EMI shielding effectiveness of ∼61 dB. Notably, the CMA also maintains outstanding structural stability and EMI performance under extreme conditions (−196°C to 1300°C). These superior combined properties indicate that the CMA is an optimal candidate for thermal insulation and EMI shielding materials for use in demanding applications such as aerospace and building.

## RESULTS AND DISCUSSION

### Preparation and characterization of the CMA

Ti_3_C_2_T*_x_* MXene nanosheets were synthesized by selectively etching the Al layer from the Ti_3_AlC_2_ MAX phase. X-ray diffraction (XRD) patterns reveal that the 104 peak of Ti_3_AlC_2_ MAX (∼39°) is absent in the MXene, confirming successful removal of the Al element during the etching process (Fig. S4a). As shown in Fig. S4b–e, the obtained MXene nanosheets exhibit a lateral size of ∼19.6 μm and a thickness of ∼1.5 nm, as determined by scanning electron microscope (SEM) imaging and atomic force microscope (AFM) imaging. Transmission electron microscope (TEM) imaging and selected-area electron diffraction patterns reveal the MXene nanosheets possess high crystallinity hexagonal structures without defects (Fig. S4f) [37]. In addition, the obtained montmorillonite (MMT) nanosheet building blocks have a thickness of ∼1.3 nm and a lateral size of ∼280 nm, while the cellulose nanofiber (CNF) building blocks exhibit a diameter of ∼4 nm and a length of ∼0.9 μm (Fig. S5).

**Figure 1.**
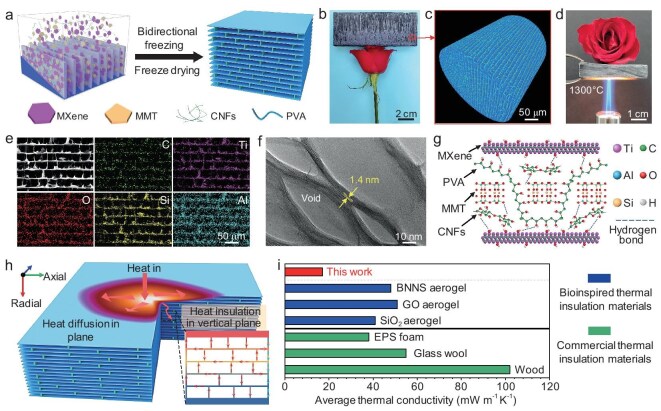
Fabrication and structural characterization of the CMA. (a) Schematic illustration of the fabrication of the CMA via bidirectional freeze casting. (b) Digital photograph showing a large-scale CMA placed on the flower to demonstrate its light weight. (c) 3D reconstruction of the CMA from a micro-CT image. (d) Photograph indicating that the CMA with a thickness of 1 cm can protect the rose flower for at least 1 min under the thermal shock of a butane blowtorch (1300°C). (e) SEM image and EDS mapping of the cross-section of the CMA, showing uniform distribution of C, Ti, O, Al, Si, which confirms the cuttlebone-like wall–septa microstructure. (f) TEM image of the cross-section of the CMA, showing an ultrathin septa layer of ∼100 nm and nanopores of ∼20 nm. (g) Structure model illustrating the interfacial interactions among MXene nanosheets, MMT, PVA, and CNF building blocks. (h) Schematic illustration of the isotropic thermal insulation mechanism of the CMA. (i) Comparison of thermal conductivity among bioinspired and commercial thermal insulation materials.

Figure 1a illustrates the fabrication process of the CMA by bidirectional freeze casting. A uniform aqueous slurry was first prepared by mixing 2D MXene and MMT nanosheets, CNFs, and polyvinyl alcohol (PVA) molecules. Subsequently, the homogeneous slurry was poured into a square polydimethylsiloxane mold, placed on a steel plate, and then directionally frozen at −100°C. The ice crystals grew directionally along the temperature gradient, compressing the solute into an oriented structure [38]. After removing the ice crystal template via freeze-drying, we obtained a large scale of CMA exceeding 12 cm × 5 cm × 1 cm (Fig. S6). As shown in Fig. 1b, the CMA exhibits lightweight properties, with an ultralow density of 8.5 mg cm^−3^, demonstrated by using a rose flower to support a large piece of CMA with a volume of 60 cm^3^. Three-dimensional (3D) reconstruction was used to observe the surface and inside microstructure, and the X-ray micro-computed tomography (micro-CT) image reveals the CMA with a long-range ordered porous structure (Fig. 1c, Fig. S7, and Movie S1). Figure 1d and Movie S2 demonstrate that the CMA can protect a rose flower for at least 1 min under the thermal shock of a butane blowtorch (1300°C), highlighting its exceptional fire resistance and thermal insulation performance. This is attributed to the CMA having high porosity (∼98%) and a well-defined wall–septa like hierarchical porous architecture, as well as a large septa layer spacing of ∼50 μm.

Compared to the typical lamellar porous structure without bridges of pure MXene aerogel, SEM images and X-ray spectroscopy (EDS) mapping reveal that the CMA features dendrites and bridges (wall) connecting the stacked 2D nanosheets layer (septa) after cross-linking of the viscous PVA molecules and the CNFs nanofibers, as shown in Fig. 1e, Fig. S8a–d, and Table S1. In addition, the number of walls connecting the septa gradually increases with the density of the CMA (Fig. S8e, f). The TEM results indicate that the septa of the CMA with an ultrathin thickness of ∼100 nm, with MMT and CNF nanomaterials embedded within the MXene layers (layer spacing 1.4 nm), forms pores of ∼20 nm. This hierarchical structure further extends the path of solid/air conduction and convection, thereby enhancing thermal insulation performance (Fig. 1f and Fig. S9) [21]. The Fourier transform infrared (FTIR) spectroscopy of pure MXene aerogel and CMA are presented in Fig. S10, the peaks at 1054 cm^−1^ and 1394 cm^−1^ are attributed to the stretching vibration of C–O and –OH groups in MXene. The band corresponding to the stretching vibration of –OH shifts to a lower wavenumber (from 3453 cm^−1^ to 3435 cm^−1^), demonstrating the strong hydrogen bonding interactions between the MXene, MMT, CNFs, and PVA [39]. As displayed in Fig. S11, X-ray photoelectron spectroscopy (XPS) reveals an increase in the O element and the appearance of Si 2p in the spectra for CMA compared to pure MXene aerogel, further indicating the hydrogen bonding interface interactions. In addition, the thermogravimetric curve demonstrates the excellent thermal stability of the CMA, as shown in Fig. S12. The structural model of the CMA is shown in Fig. 1g, where CNFs and PVA act as bridges, tailoring the interactions between surface chemical groups and preventing the restacking of MXene and MMT nanosheets.

Figure 1h illustrates the multiscale thermal insulation mechanism of the CMA. At the macroscale, the CMA consists of ∼98% pores and only ∼2% solid constituent, significantly limiting air conduction and convection. At the micro/nanoscale, the large interlayer spacing between the wall–septa not only effectively reduces heat conduction in the solid phase but also contains nanopores much smaller than the mean free path of air molecules, thereby restricting thermal convection [18]. Furthermore, this hierarchical structure provides complex pathways that further block heat conduction between the solid and gas phases, providing heat diffusion in the horizontal plane (axial) and heat insulation in the vertical plane (radial). As a result, the obtained CMA demonstrated an ultralow average thermal conductivity (18.4 mW m^−1^ K^−1^) in both the radial and axial directions, outperforming other bioinspired thermal insulation materials (e.g. boron nitride (BNNS) aerogel [22], graphene oxide (GO) aerogel [40], and silica (SiO_2_) aerogel [41]) and commercial thermal insulation materials (e.g. expanded polystyrene (EPS) foam, glass wool [42], and wood [42]), as shown in Fig. 1i and Table S2.

### Mechanical and sensing performance of the CMA

**Figure 2.**
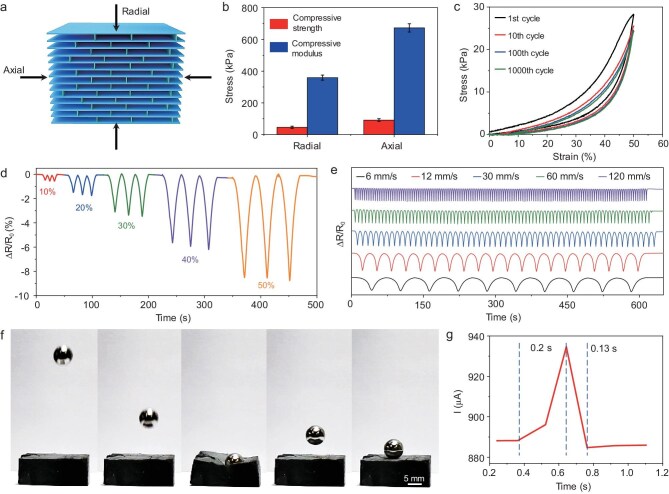
Mechanical and sensing performance of the CMA. (a) Schematic diagram of the compressive test of the CMA in both radial and axial directions. (b) Compressive strength and modulus of the CMA in the radial and axial directions. (c) Compressive stress-strain curves of the CMA under 1000 compression cycles with a maximum strain of 50% in the radial direction. (d) Relative resistance changes of the CMA under different compressive strains (radial direction). (e) Relative resistance changes of the CMA at various compressive rates under 30% strain (radial direction). (f) A series of real-time images from a high-speed camera showing that CMA rebounds a solid steel ball at high speed. (g) Response and recovery time of the CMA at a compressive rate of 500 mm s^−1^ under 10% strain.

The CMA exhibits excellent compressibility, fatigue resistance, and sensing performance. Figure 2a and Fig. S13 show the compressive stress-strain curves of the CMA along with the radial and axial direction. The CMA embodies a stiff distinct deformation behavior in the axial direction, while in the radial direction high reversible compressibility and an elastic deformation behavior is observed. We obtain an ultrahigh compressive strength of 51 kPa and compressive modulus of 373 kPa in the radial direction, alongside a superhigh compressive strength of 105 kPa and compressive modulus of 712 kPa in the axial direction (Fig. 2b). As shown in Fig. S14 and Table S3, the compressive strength in both radial and axial directions are superior to reported MXene aerogels [39,43–49]. This is due to the vertical walls connecting the septa effectively distributing stress concentrations in the septa during radial compression of the CMA. While under axial compression, the continuous septa structures of the CMA bear the primary load. Impressively, the CMA can endure over 1000 compression cycles at a strain of 50%, showcasing excellent dynamic fatigue resistance in the radial direction (Fig. 2c).

Owing to the excellent electrical conductivity of the MXene nanosheets, the CMA can also function as a strain sensor, demonstrating rapid and stable electrical response capabilities. As illustrated in Fig. 2d and Fig. S15, the relative resistance changes of the CMA sensor decrease linearly with increasing compressive strain under uniaxial compression ranging from 10% to 50% (radial direction). This behavior is attributed to the increased contact area between adjacent septa, which enhances the conductive pathways after compression. At the same time, the CMA sensor also exhibits a stable relative resistance change under varying frequencies at a compressive strain of 30% (radial direction), as well as during 1000 compression cycles, underscoring its outstanding fatigue resistance and reproducibility (Figs 2e and S16).

Furthermore, we have evaluated the impact resistance of the CMA. When a solid steel ball weighing 10 g (100 times heavier than the aerogel sample) was dropped from a height of 300 mm, the CMA successfully rebounded the ball while maintaining its structural integrity without any cracking (Fig. 2f and Movie S3). Notably, the CMA sensor showed a fast response and recovery speed at a compressive rate of 500 mm s^−1^ under 10% strain, with a response time of only 0.20 s and recovery time of only 0.13 s, indicating real-time responsiveness to changes in pressure (Fig. 2g). As shown in Fig. S17 and Table S4, the CMA sensor demonstrates superior performance compared to conventional MXene aerogel and hydrogel sensors [39,47,50–55]. This enhancement can be attributed to the CMA having a mass of wall to bridge the septa, forming more conductive pathways by reducing pore distance and increasing the number of wall–septa connections under external force.

### Thermal insulation performance of the CMA

**Figure 3.**
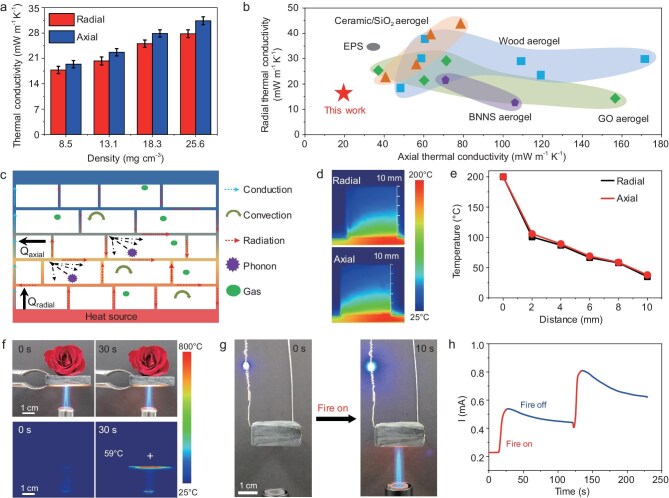
Thermal insulation properties of the CMA. (a) Thermal conductivity of the CMA in both radial and axial directions. (b) Comparison of the radial and axial thermal conductivity of biomimetic MXene aerogel with other reported thermal insulation materials. (c) Schematic illustration of the thermal insulation contributions in the radial and axial directions of the CMA with a wall–septa-like microstructure. (d) Real-time infrared images of the CMA in both radial and axial directions after being placed on a 200°C hot plate for 10 min. (e) Temperature distribution curves of the CMA in both radial and axial directions at different heights. (f) Real-time optical (top) and infrared (bottom) images of a rose flower placed on the CMA under the thermal shock of a butane blowtorch (1300°C). (g) Digital photographs showing a fire-warning smart switch that lights up the LED when the CMA is exposed to fire. (h) Response curves of the CMA upon fire on/off treatment.

Aerogels are widely recognized as the best thermal insulation materials due to their low thermal conductivity and high porosity, and play a critical role in aerospace, construction, and other fields. Thanks to its wall–septa microstructure, the CMA exhibits superior isotropic thermal insulation properties, with thermal conductivity rising as the density increases (Fig. 3a). For instance, a CMA with a density of 8.5 mg cm^−3^ demonstrates extremely low thermal conductivity in both radial and axial directions, measuring 17.1 mW m^−1^ K^−1^ and 19.7 mW m^−1^ K^−1^, respectively. As shown in Fig. 3b and Table S5, the average thermal conductivity (18.4 mW m^−1^ K^−1^) is significantly lower than that of commercial and bioinspired aerogels, representing the lowest value reported to date [22,34,40–42,56–66]. Figure 3c reveals the contributions of conduction, convection, radiation, phonon, and gas molecules to the ultralow thermal conductivity of the CMA in both radial and axial directions. The high porosity of the CMA effectively reduces air conduction and convection, while blocking phonon conduction pathways, further diminishing solid/air interface phonon scattering [67]. Simultaneously, the unique wall–septa microstructure of the CMA provides consistent and complex pathways in both axial and radial directions, reducing solid/gas heat conduction and convection. Additionally, the wall–septa structure generates both multiple reflection and layered air-blocking effects, significantly lowering thermal conductivity in both radial and axial directions [18]. More importantly, the abundant presence of nanopores is a critical factor contributing to the low thermal conductivity of the CMA. According to the Knudsen effect, when the size of nanopores approaches the mean free path of gas molecules in the air (∼70 nm), it restricts the movement of gas molecules, thereby reducing thermal conductivity [68]. CMAs with low densities, and a nanopore structure of ∼20 nm can simultaneously minimize heat conduction in solids, and reduce conduction and convection in air, collectively enabling exceptional thermal superinsulation performance in both axial and radial directions.

The thermal insulation performance of the CMA was further analyzed through its thermal response using a hot plate up to 200°C as the heat source. Figure 3d, e displays similar thermal infrared images of the CMA both in radial (top) and axial (bottom) directions after 10 min, with the average temperature at different heights being nearly identical, indicating isotropic thermal insulation performance. The CMA also demonstrates excellent thermal insulation behavior at small thicknesses, evidenced by the surface temperature decreasing from ∼56.6°C at 5 mm aerogel thickness to ∼28.8°C at 25 mm, as shown in Fig. S18. In addition, the CMA with a 10 mm thickness maintains long-term thermal insulation stability, with a surface temperature of 44.1°C after 60 min (Fig. S19). Based on its exceptional thermal insulation properties, we demonstrated the application of the CMA in materials for building insulation. As shown in Fig. S20a, a 5 mm thick CMA was attached to the roof of a house model. Compared to the house model without aerogel, the indoor temperature (T2) of the aerogel-covered model did not significantly increase when exposed to sunlight after 300 s, maintaining a comfortable temperature of ∼26°C (Fig. S20b, c). It is worth noting that roof temperature (T1) with the aerogel reached up ∼67°C, which was ∼19°C higher than that without aerogel. Moreover, when exposed to an 808 nm near-infrared (NIR) laser with power densities ranging from 0.28 W cm^−2^ to 7.04 W cm^−2^, the surface temperature of the CMA increased significantly from ∼26°C to ∼631°C, demonstrating excellent photothermal conversion capability and thermal stability (Fig. S21).

In addition, the CMA also exhibits robust fire resistance capabilities. As shown in Fig. 3f and Movie S4, a 1 cm thick CMA effectively protected a rose flower, keeping it fresh under the thermal shock of a butane blowtorch (1300°C) after 30 s. Real-time infrared images reveal that the top surface temperature of the CMA maintained relatively low at only 59°C. It should be noted that the inspection limit of the infrared thermal imager is 800°C. As shown in Fig. S22, the CMA retains an intact wall–septa microstructure despite the thermal shock at 1300°C. Meanwhile, many nanoparticles are observed on the wall/septa surfaces, which are attributed to the combustion and subsequent oxidation of MXene nanosheets into TiO_2_. Moreover, as evidenced by the XPS results (Fig. S23), the intensity of the TiO_2_ peak in the CMA after thermal shock is significantly higher than that of the untreated CMA, as well as the C-C peak, indicating that CMA surface carbonization occurs during the initial thermal shock stage. These results indicate the remarkable high-temperature thermal insulation and exceptional working temperature of CMA. Therefore, we developed a fire-warning smart switch to extend the practical application of the CMA. Upon exposure to fire, the light-emitting diode (LED) connected to the CMA illuminates significantly brighter within 10 s (Fig. 3g). This rapid response is attributed to surface carbonization of the CMA, which reduces its electrical resistance and increases the current (I) upon exposure to high temperature flames. The fire-warning smart switch can be triggered again with repeated ignition, showcasing outstanding reusability and durability, as shown in Fig. 3h and Movie S5. These results highlight that the CMA combines thermal insulation with structural stability, offering great potential for application in the thermal insulation field under high temperature environments. These include thermal protection materials in aerospace vehicles and smart fire-warning systems in building.

### Electromagnetic interference shielding performance of the CMA

**Figure 4.**
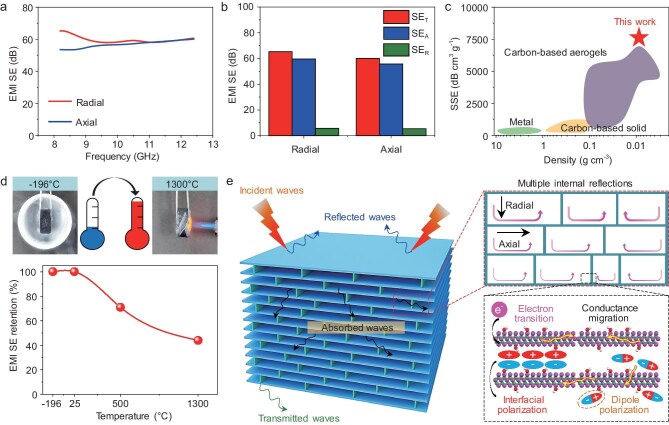
Electromagnetic interference shielding properties of the CMA. (a) EMI SE values of the CMA with a density of 8.5 mg cm^−3^ in both radial and axial directions. (b) Total EMI SE (SE_T_), its absorption (SE_A_), and reflection (SE_R_) of the CMA in both radial and axial directions at a frequency of 8.2 GHz. (c) Comparison of specific shielding effectiveness (SSE) versus density between the CMA and other reported EMI shielding materials. (d) EMI SE values of the CMA in the radial direction after treatment at −196°C, 25°C, 500°C, and 1300°C for 1 min. (e) Schematic illustration of the EMI shielding synergistic mechanisms of the hierarchical porous architecture of the CMA.

Modern communication technology and aerospace electronic equipment are often exposed to electromagnetic waves, which can severely disrupt their normal operation. High-performance EMI shielding materials not only effectively prevent the leakage of electromagnetic information but also mitigate the health risks posed to humans by electromagnetic radiation [69]. Benefiting from the high electrical conductivity of MXene nanosheets, the CMA is considered an ideal candidate for electromagnetic interference shielding effectiveness (EMI SE). As shown in Fig. S24, the EMI SE performance of a 10 mm thick CMA was evaluated in the frequency range of 8.2 GHz to 12.4 GHz (X-band). The EMI SE of the CMA primarily depends on the its density, increasing from ∼61 dB for 8.5 mg cm^−3^ to ∼89 dB for 25.6 mg cm^−3^. Simultaneously, the CMA with a density of 8.5 mg cm^−3^ shows a high EMI SE of ∼61 dB in the radial direction and ∼58 dB in the axial direction, indicating isotropic EMI shielding performance (Fig. 4a). This is attributed to the hierarchical porous structures of the CMA, which effectively induces multi-reflection of electromagnetic waves at the wall (axial direction) and void-cell of septa (radial direction) interfaces. The corresponding calculated total EMI SE (SE_T_), microwave absorption (SE_A_), and microwave reflection (SE_R_) of the CMA (8.5 mg cm^−3^) are displayed in Fig. 4b. The SE_A_ (∼57 dB in the radial direction and ∼54 dB in the axial direction) are significantly higher than the SE_R_ values (∼4 dB in both radial and axial directions), suggesting that the primary EMI shielding mechanism inside the CMA is based on the absorption of electromagnetic waves. In addition, the power coefficient absorptivity (*A*), reflection (*R*), and transmission (*T*) of the CMA were analyzed to further evaluate the ability of EMI shielding to absorbed, reflected, and transmitted electromagnetic waves, respectively. As shown in Fig. S25, the power coefficient of *R* of the CMA is 0.85 in the radial direction and 0.84 in the axial direction, which is higher than the *A* values (0.15 in the radial direction and 0.16 in the axial direction) and *T* (0). These results suggest that the CMA mainly functions as a reflection-based electromagnetic shielding material rather than an absorption-based one.

Density is a critical parameter influencing the EMI SE performance of shielding materials. As shown in Fig. S26, Fig. 4c, and Table S6, the lightweight CMA at low density exhibits excellent EMI SE performance and achieves an ultrahigh SSE of ∼7177 dB cm^3^•g ^−1^ at a density of 8.5 mg cm^−3^, which surpasses metal and carbon-based solid/aerogels [43,46,70–75]. Building on its extraordinary performance, including compressibility, fatigue resistance, and exceptional working temperature, the CMA demonstrates excellent durability and stability under extreme conditions. After 1000 compression cycles at a strain of 50%, the EMI SE performance of the CMA in the radial direction was increased to ∼117% (Fig. S27). This improvement is attributed to the septa coming into close proximity, enhancing conductive pathways and facilitating the migration and hopping of electrons, as illustrated in Fig. S24. In addition, the CMA maintains stable EMI SE performance across a wide temperature range from −196°C to 1300°C, as shown in Fig. 4d and Fig. S29. Notably, the CMA shows almost no decline in EMI SE performance after treatment for low temperature (−196°C) in liquid nitrogen. The CMA shows >70% EMI SE retention and maintains an intact microtopography structure after treatment at 500°C with an alcohol lamp, while the EMI SE retention still has 44% after treatment at 1300°C (Fig. S30). Consequently, the outstanding EMI SE performance and structural stability across a wide temperature range (−196°C to 1300°C) of CMA shows an important potential to be applied for aerospace and electronic devices operating under extreme conditions.

The hierarchical porous architectures endow the CMA with ultrahigh EMI SE performance that is attributed to multiple shielding mechanisms of reflection and absorption at abundant interfaces, as is shown in Fig. 4e. Specifically, the MXene nanosheets provide an efficient conductive network that enables reflection shielding at the interface. Additionally, the high porosity of the MXene aerogel creates significant impedance mismatch between the air and the highly conductive material interface, leading to partial reflection of incident electromagnetic waves [76]. Moreover, the numerous heterogeneous interfaces between conductive MXene, MMT, CNFs, and PVA, as well as the hierarchical wall–septa structure, induce multiple internal reflections and scattering, forming longer propagation paths and resulting in greater electromagnetic energy dissipation [77]. Furthermore, the MXene nanosheets generate ohmic losses (interfacial polarization loss and conductivity loss), effectively converting electromagnetic energy into thermal energy and attenuating microwaves. In addition, localized imperfections and terminal groups, such as –O–, –F, and –OH, on the surfaces of MXene induce an uneven distribution of charge density, creating local dipoles under the electromagnetic field leading to increasing polarization loss [78]. Therefore, the synergistic combination of reflection, absorption, and multiple internal scattering or ohmic losses enables the CMA to effectively shield electromagnetic waves, exhibiting excellent EMI shielding performance.

## CONCLUSION

In summary, we have successfully developed a cuttlebone-inspired lightweight and isotropic thermal insulation MXene aerogel with exceptional resistance to extreme conditions. By employing bidirectional freeze casting technology, we engineered the MXene aerogel with a wall–septa like microstructure, achieving remarkable thermal insulation properties with ultralow thermal conductivity values of 17.1 mW m^−1^ K^−1^ in the radial direction and 19.7 mW m^−1^ K^−1^ in the axial direction. Furthermore, the CMA demonstrates rapid sensing response capabilities, excellent fire resistance, and superior EMI shielding effectiveness of ∼61 dB. Remarkably, the CMA maintains outstanding structural integrity and EMI shielding performance stability across a wide temperature range from −196°C to 1300°C. These exceptional properties collectively establish the CMA as an ideal candidate for advanced thermal insulation and EMI shielding applications in demanding environments, including aerospace, electronic devices, and building materials.

## METHODS

### Materials

Ti_3_AlC_2_ MAX phase powder (∼200 mesh) was purchased from Jilin 11 Technology Co., Ltd. Lithium fluoride (LiF) was obtained from Shanghai Maclin Biochemical Technology Co., Ltd. Hydrochloric acid (HCl, GR, 36%–38%) was supplied by Sinopharm Chemical Reagent Co., Ltd. Polyvinyl alcohol (PVA) (Mw 13 000–23 000, 87%–89% hydrolyzed) was purchased from Greagent. Montmorillonite (MMT) was acquired from Nanocor. Polydimethylsiloxane (PDMS, Sylgard 184 silicon elastomer) was provided by Dow Corning. Cellulose nanofibers (CNFs) were obtained from Zhongshan Nano Fiber Co., Ltd. All materials were used as received without further purification. The deionized water used in the experiment was prepared in the laboratory.

### Characterization

Scanning electron microscope (SEM) images were captured using a field emission scanning electron microscope (Quanta 250FEG) at an acceleration voltage of 10 kV. X-ray micro-computed tomography (Zeiss) was employed to image the CMA sample (3 mm × 3 mm × 3 mm) with a voxel size of 1 µm. Transmission electron microscope (TEM) images were acquired using a JEM-2100F instrument with an acceleration voltage of 200 kV. X-ray spectroscopy (EDS) mapping was obtained using an Oxford instrument (INCA Energy 250). Atomic force microscope (AFM) images were obtained using a Bruker MultiMode8 in Smart Scan mode. X-ray diffraction (XRD) patterns were recorded using a XRD Bruker D8 Advance with Cu-Kα radiation and a scanning speed of 2° min^−1^. Fourier-transform infrared spectroscopy (FTIR) spectra were collected by a INX10 spectroscope (Thermo Scientific). Thermogravimetric analysis (TGA) was conducted in a nitrogen atmosphere at a heating rate of 10°C min^−1^ from room temperature to 800°C using a STA449F5 instrument (Netzsch). X-ray photoelectron spectroscopy (XPS) spectra were collected using an ESCALABXi+ (Thermo Scientific) with a monochromatic Al-Kα X-ray source. The mechanical compression tests were conducted using a universal testing machine (EM6.103-T, 1KN), and the sensing performance of the CMA was evaluated using a Keithley 2400 DC power supply. Thermal conductivity of the CMA in both the axial and radial directions was measured using a hot disk thermal constant analyzer (TPS 2500S) on samples with dimensions of 10 mm × 10 mm × 10 mm. Infrared thermal images and videos were recorded using a FOTRIC 228s infrared thermal imager when thermal shock and infrared laser were applied.

## Supplementary Material

nwaf342_Supplemental_Files
