# Supplementary material for: Isotropic thermal insulating cuttlebone-inspired MXene aerogel
Source: Natl Sci Rev. 2025 Aug 25;12(10):nwaf342. doi: 10.1093/nsr/nwaf342 (PMC12485986; doi:10.1093/nsr/nwaf342)
Supplement: nwaf342_Supplemental_Files [file nwaf342_supplemental_files.zip › Supplementary data.pdf]

Supplementary Materials for

Isotropic thermal insulating cuttlebone-inspired MXene aerogel

Junsong Fu[1,2,3,†], Wangwei Lian[1,2,3,†], Yankang Deng[1,2,3], Zixuan Fang[1,2,3] and Qunfeng Cheng[1,2,3,4*]

*Corresponding author: Qunfeng Cheng
E-mail: chengqf@ustc.edu.cn.

This file includes:

Supplementary text

Figures S1 to S30

Tables S1 to S6

Other supplementary materials for this manuscript include the following:

Movies S1 to S5

Supplementary text

Synthesis of $Ti_3C_2T_x$ MXene nanosheets

The $Ti_3C_2T_x$ MXene nanosheets were synthesized using a minimally intensive layer delamination method. Specifically, 4.8 g of lithium fluoride (LiF) was added to 60 mL of 9 M hydrochloric acid (HCl) solution and dissolved. Then, 3 g of Ti_3AlC_2 MAX powder was added to the mixture and stirred at 50°C for 30 hours. After the reaction was completed and the mixture cooled to room temperature, the resulting product was washed 7 to 9 times with deionized water and centrifuged at 3500 rpm for 5 min each time until the pH of supernatant was exceeded 6. Subsequently, the dispersion was continuously vibrated for 5 min, followed by centrifuging at 1500 rpm for 30 min separate the supernatant. The remaining sediment underwent several cycles of vibration for 5 min and centrifuged at 1500 rpm for 30 min to extract additional supernatant. This process was repeated until the supernatant turned light green. Next, the collected dark green supernatant was centrifuged at 4500 rpm for 20 min to obtain the sediments containing the $Ti_3C_2T_x$ MXene nanosheets. Finally, the $Ti_3C_2T_x$ MXene nanosheets dispersion was obtained by diluting the sediment with deionized water to achieve a concentration of 10 mg ml^{-1} and stored at 4°C until further use.

Preparation of cuttlebone-inspired MXene aerogel (CMA) via bidirectional freeze casting

A slurry was prepared by mixing an aqueous suspension $Ti_3C_2T_x$ MXene nanosheets with montmorillonite (MMT), polyvinyl alcohol (PVA), and cellulose nanofibers (CNFs) aqueous solution at a mass of 3:1:1:1, followed stirring for at least 1 h. After defoaming using a vacuum pump , the MXene/MMT/PVA/CNFs slurry was poured into a polydimethylsiloxane (PDMS) mold placed on the surface of a steel plate and directionally frozen at -100°C. Once the slurry was completely frozen, ice within biomimetic MXene aerogel was sublimated through freeze drying at -80°C under a pressure of 1 Pa for 96 h.

Porosity calculation

First, the scaffold density (ρ_s) of the CMA was determined. The scaffold density is the weighted average density of each component, the scaffold density was calculated using the following formula (1):

$$\rho_s = \frac{\Sigma(m_i \cdot \rho_i)}{\Sigma m_i} \tag{1}$$

where m_i is the mass fraction of the i-th component, and ρ_i is the density of the i-th component. The theoretical densities of the ρ_{MXene}、ρ_{MMT}、ρ_{CNFs}、ρ_{PVA} are 3.68 g cm^{-3}, 2.6 g cm^{-3}, 1.5 g cm^{-3}, 1.3 g cm^{-3}, respectively.

Next, the bulk density (ρ_b) of the CMA was calculate using the following formula (2):

$$\rho_b = \frac{m}{V} \tag{2}$$

where m is the mass of the CMA sample, and V is the volume of the biomimetic MXene aerogel sample.

Finally, the porosity (P) of the CMA sample was calculated using the following equation (3):

$$P = 1 - \frac{\rho_b}{\rho_s} \tag{3}$$

Measurement of thermal conductivity

The thermal conductivity of the CMA was determined using the transient plane source method. Specifically, a Hot Disk thermal conductivity analyzer (TPS 2500S) probe was placed on the surface of the CMA sample. When electric current passes through the nickel probe, it generates a temperature rise. The resulting heat simultaneously diffuses into the sample on both sides of the probe. By recording the temperature and the probe's response time, the thermal conductivity of the CMA was calculated. The axial and radial thermal conductivities were obtained by changing the direction of the aerogel sample, At least three tests are conducted in each direction, and the sample size is 10 mm × 10 mm × 10 mm.

Electromagnetic interference shielding measurement and calculation

Electromagnetic interference (EMI) shielding testing was conducted in the frequency range of 8.2−12.4 GHz using a coaxial method in a vector network analyzer (VNA, ZNA43, ROHDE&SCHWARZ). The CMA sample with the dimensions of 23 mm × 10 mm × 10 mm. The electromagnetic shielding effectiveness (EMI SE) can be calculated from the measured scattering parameters. EMI SE is defined as the logarithmic ratio of incoming power (P_I) to transmitted power (P_T) as (4):

$$EMI\ SE = 10 \log\left(\frac{P_I}{P_T}\right) \tag{4}$$

When an electromagnetic radiation is incident on the shielding material, the sum of reflection (R), absorption (A) and transmission (T) must be equal to 1, that is,

$$R + A + T = 1 \quad (5)$$

The scattering parameters (S_{22}, S_{11}, S_{21}, and S_{12}) were directly obtained from the vector network analyzer, and the corresponding reflection (R) and transmission (T) coefficients were calculated according to the following equations (6,7):

$$R = |S_{22}|^2 = |S_{11}|^2 \quad (6)$$

$$T = |S_{21}|^2 = |S_{12}|^2 \quad (7)$$

The total EMI SE (EMI SE_T) is the sum of reflection (SE_R), absorption (SE_A), and multiple internal reflections (SE_M). When the EMI SE value exceeds 15 dB, the multiple internal reflections are usually negligible. Thus, the total SE_T can be described as (8):

$$SE_T = SE_R + SE_A \quad (8)$$

where SE_R and SE_A can be calculated according to the reflection and absorption n coefficients as follows (9,10):

$$SE_R = 10\log\left(\frac{1}{1-R}\right) = 10\log\left(\frac{1}{1-|S_{11}|^2}\right) \quad (9)$$

$$SE_A = 10\log\left(\frac{1-R}{T}\right) = 10\log\left(\frac{1-|S_{11}|^2}{|S_{21}|^2}\right) \quad (10)$$

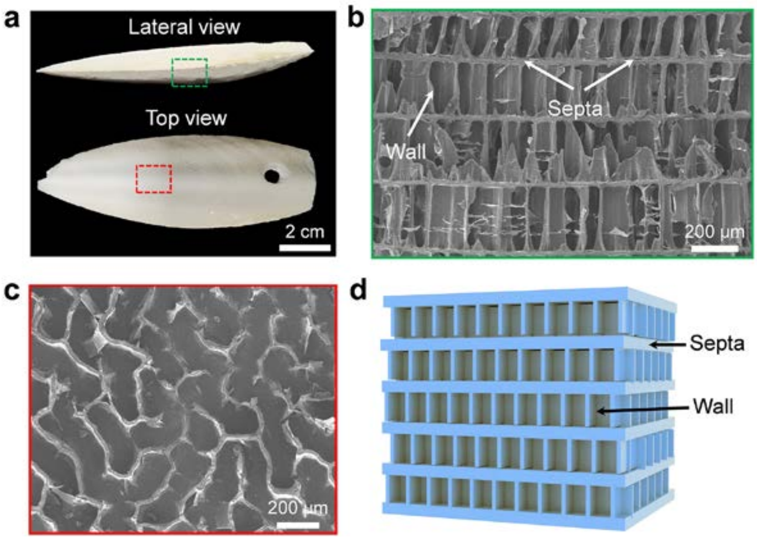

Figure S1. (a) Optical images of a cuttlebone. (b) Scanning electron microscope (SEM) image of the lateral view of cuttlebone. (c) SEM image of the top view of cuttlebone. (d) Schematic diagram of the wall–septa microstructure.

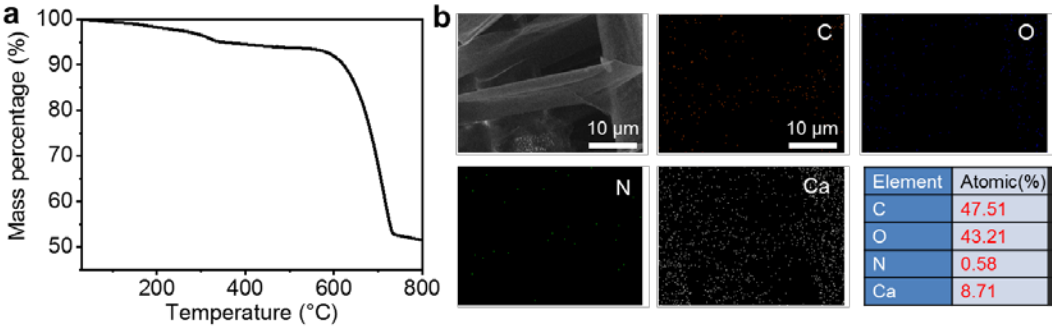

Figure S2. (a) Thermogravimetric analysis (TGA) curve of the cuttlebone. (b) SEM image and X-ray spectroscopy (EDS) mapping of the cuttlebone.

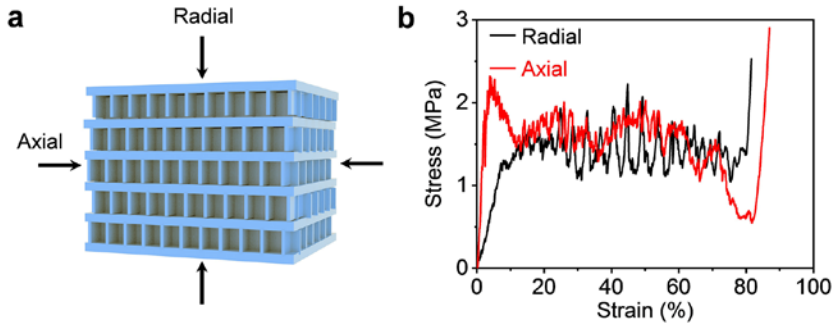

Figure S3. (a) Schematic illustration of compressive test for the cuttlebone. (b) Compressive stress–strain curves of the cuttlebone.

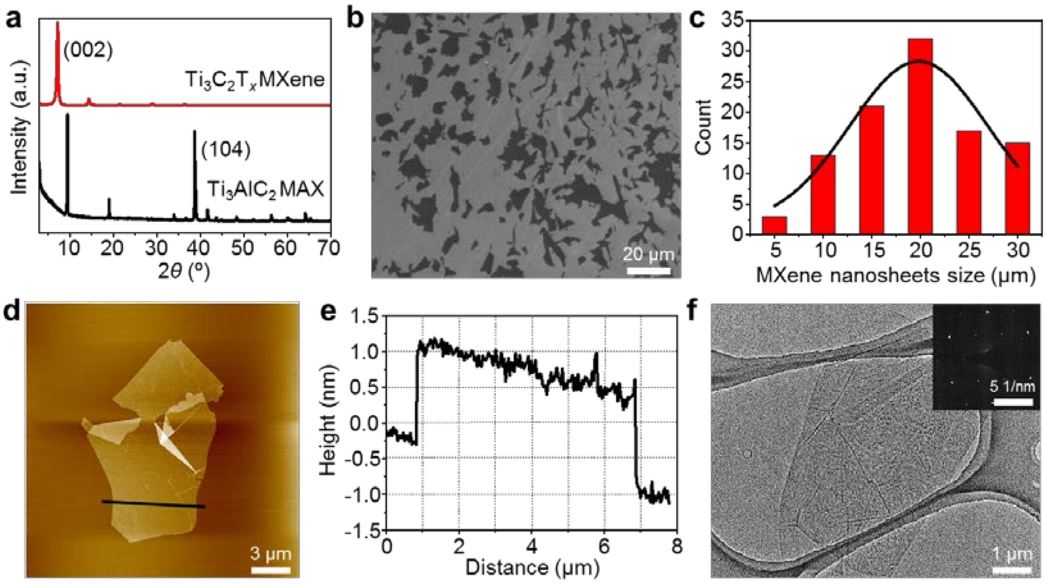

Figure S4. (a) X-ray diffraction (XRD) patterns of Ti_3AlC_2 MAX and $Ti_3C_2T_x$ MXene. (b) SEM image of $Ti_3C_2T_x$ MXene nanosheets. (c) Lateral size distribution of large $Ti_3C_2T_x$ MXene nanosheets. (d) AFM image of large MXene nanosheets. (e) The height profiles of large $Ti_3C_2T_x$ MXene nanosheets for (d) measured along the black line. (f) Transmission electron microscope (TEM) image and corresponding selective area electron diffraction pattern (inset) of $Ti_3C_2T_x$ MXene nanosheets.

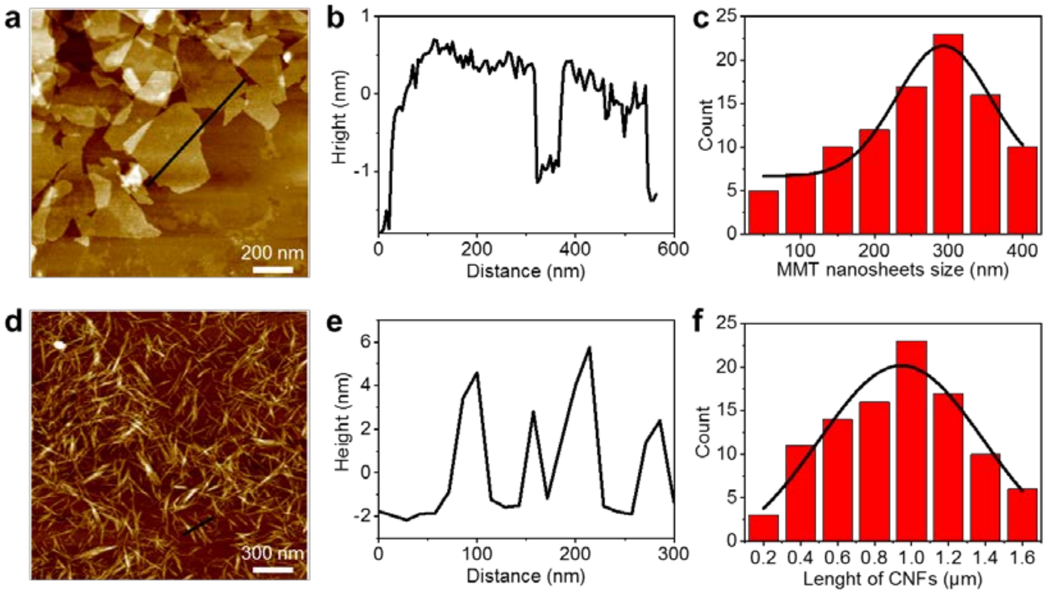

Figure S5. (a) Atomic force microscope (AFM) image of MMT nanosheets. (b) The height profiles of MMT nanosheets for (a) measured along the black line. (c) Lateral size distribution of MMT nanosheets. (d) AFM image of CNFs. (e) The height profiles of CNFs for (d) measured along the black line. (f) The length distribution of CNFs.

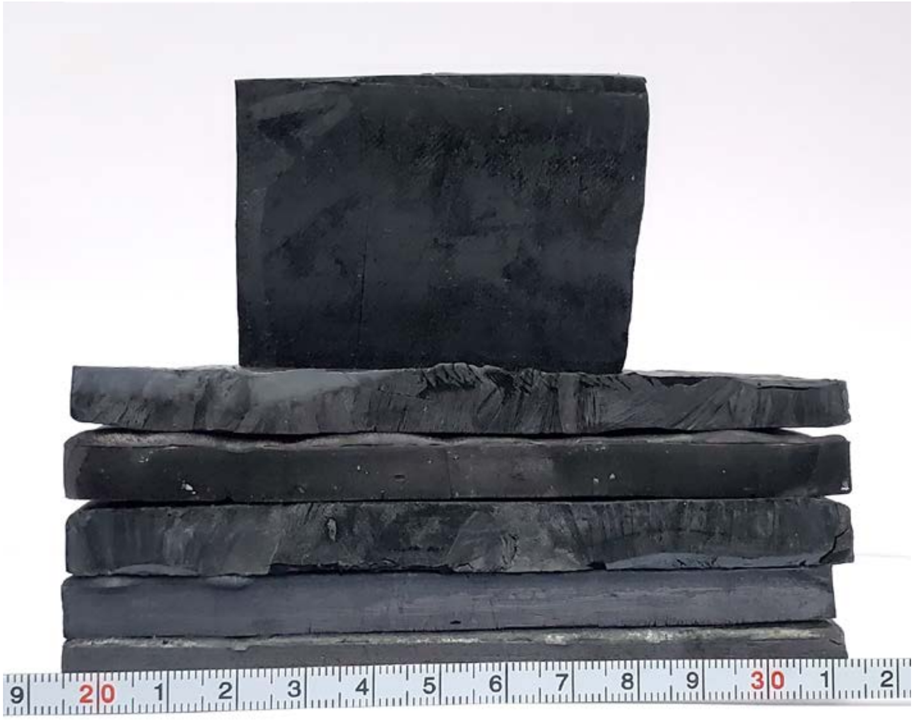

Figure S6. Photograph of the large scale CMA sample to demonstrate the scalability.

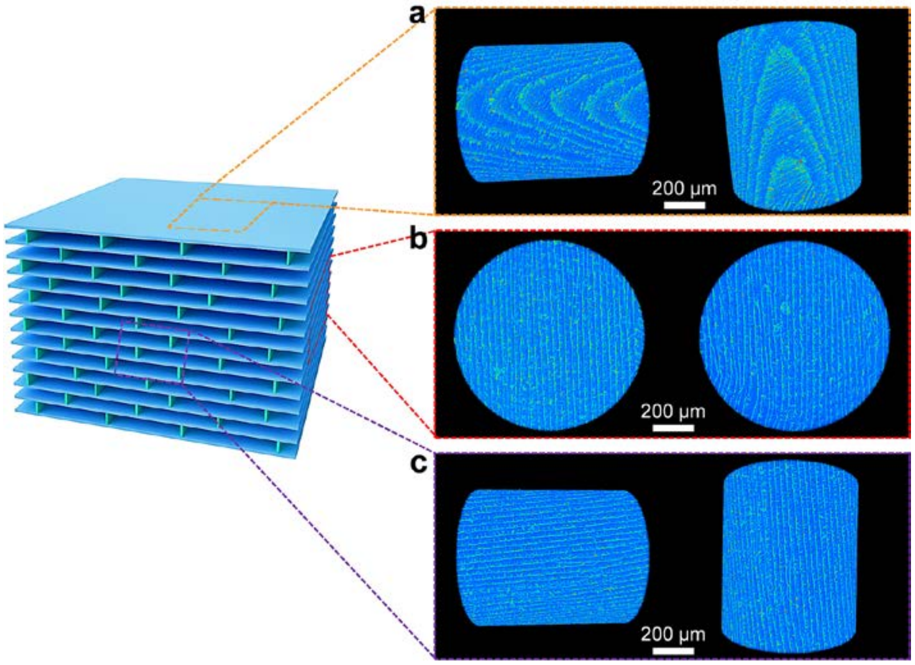

Figure S7. (a) X-ray micro-computed tomography (micro-CT) images of the top view of CMA. (b) Micro-CT images of the side view of CMA. (c) Micro-CT images of the front view of CMA.

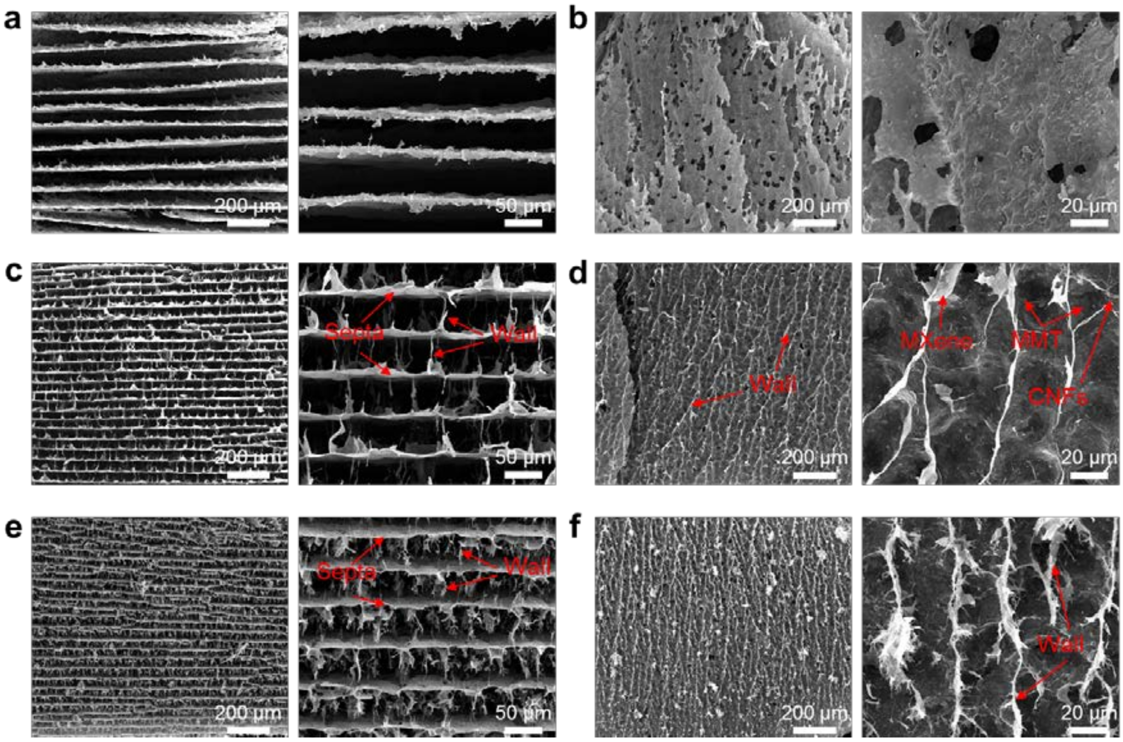

Figure S8. (a) SEM images of the cross-section of pure MXene aerogel. (b) SEM images of the top view of pure MXene aerogel. (c) SEM images of the cross-section of CMA with density of 8.5 mg cm^{-3}. (d) SEM images of the top view of CMA with density of 8.5 mg cm^{-3}. (e) SEM images of the cross-section of CMA with density of 13.1 mg cm^{-3}. (f) SEM images of the top view of CMA with density of 13.1 mg cm^{-3}.

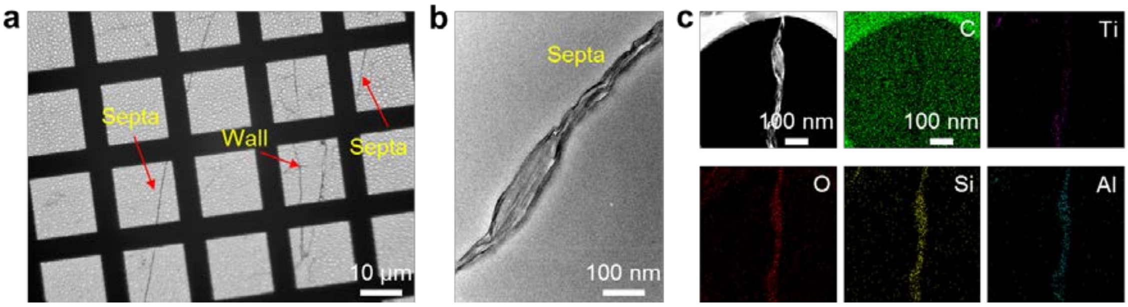

Figure S9. (a, b) TEM images of the cross-section of CMA. (c) Dark-field TEM image and corresponding energy dispersive EDS element mappings distribution of CMA.

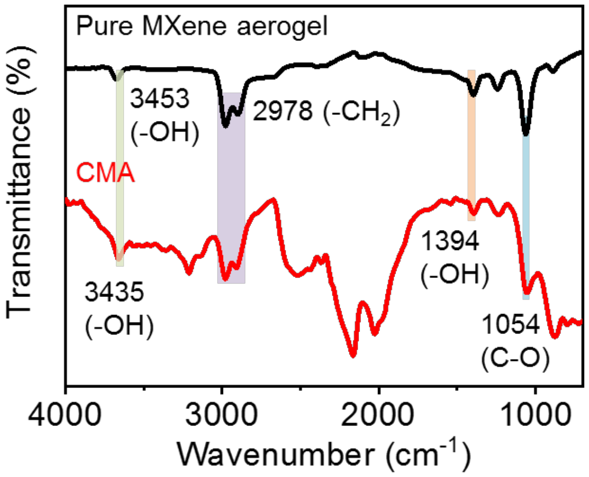

Figure S10. Fourier-transform infrared spectroscopy (FTIR) spectra of the pure MXene aerogel and CMA.

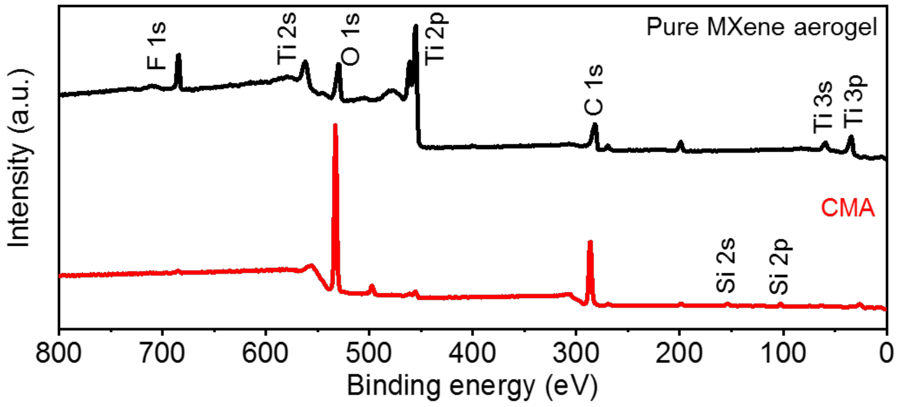

Figure S11. X-ray photoelectron spectroscopy (XPS) spectra of the pure MXene aerogel and CMA.

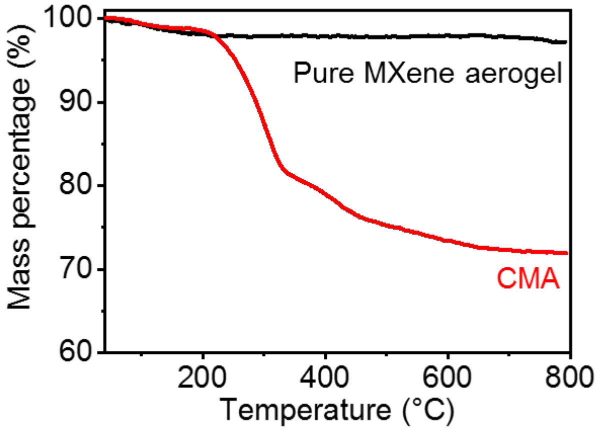

Figure S12. TGA curves of the pure MXene aerogel and CMA.

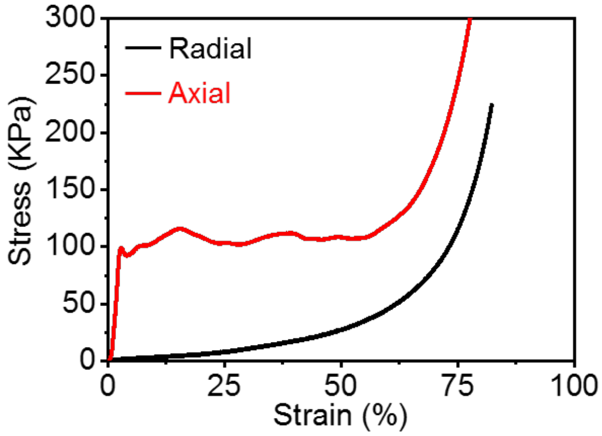

Figure S13. Compressive stress–strain curves of the CMA in both radial and axial directions.

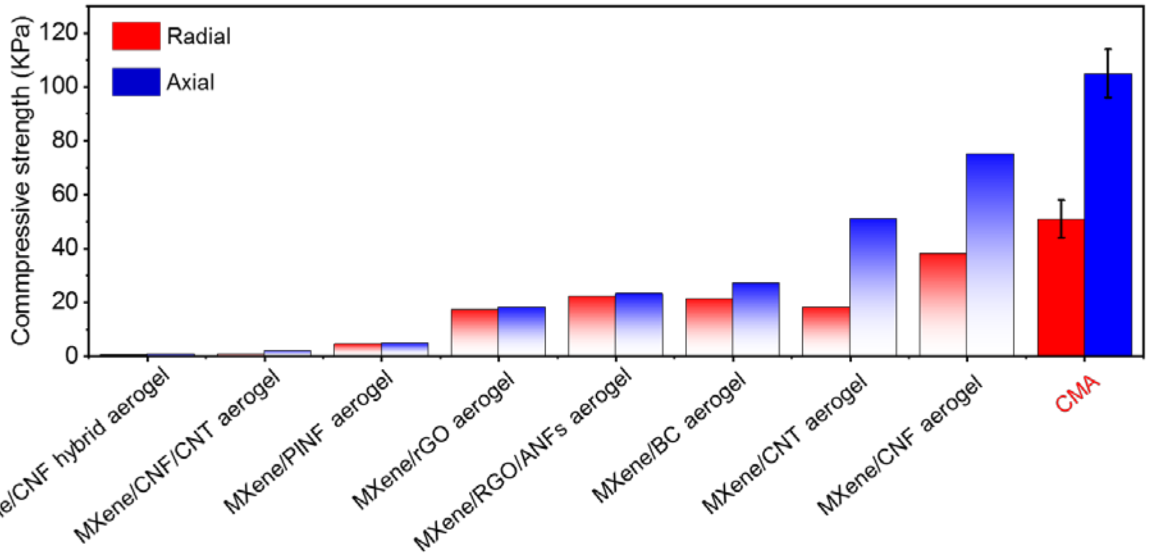

Figure S14. Comparison of the compressive strength of CMA and other reported MXene aerogels in the radial and axial directions.

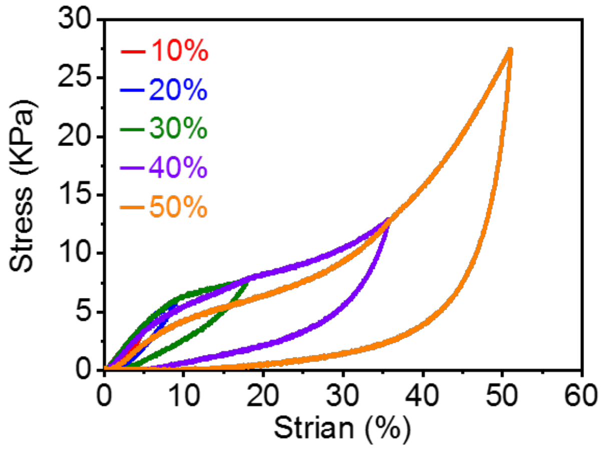

Figure S15. Uniaxial compressive stress–strain curves of the CMA (radial direction) under cyclic loading from 10% strain to 50% strain.

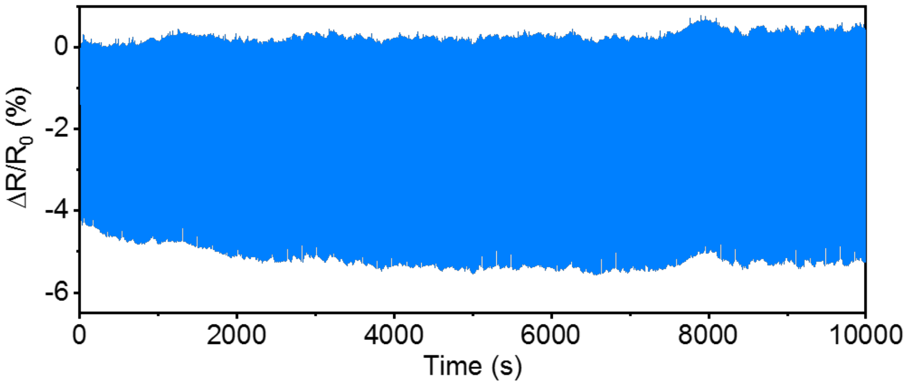

Figure S16. Relative resistance changes of the CMA with a maximum compressive strain of 30% for 1000 cycles in the radial direction.

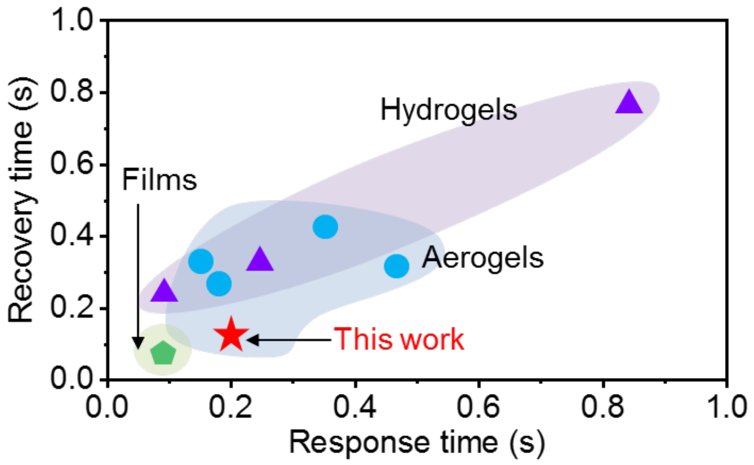

Figure S17. Comparison of the response and recovery time of CMA sensor and other MXene sensing materials.

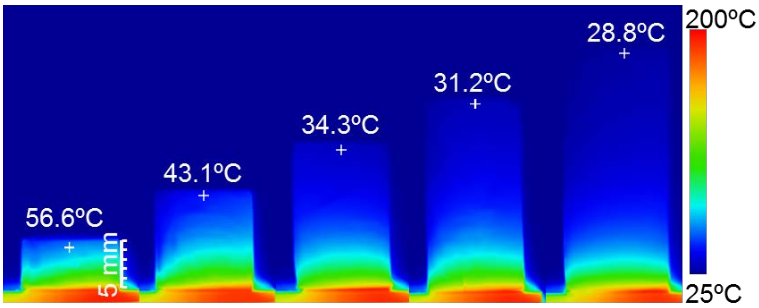

Figure S18. Real time infrared images of the CMA (radial direction) with different thickness placed on a hot plate of 200ºC after 10 min.

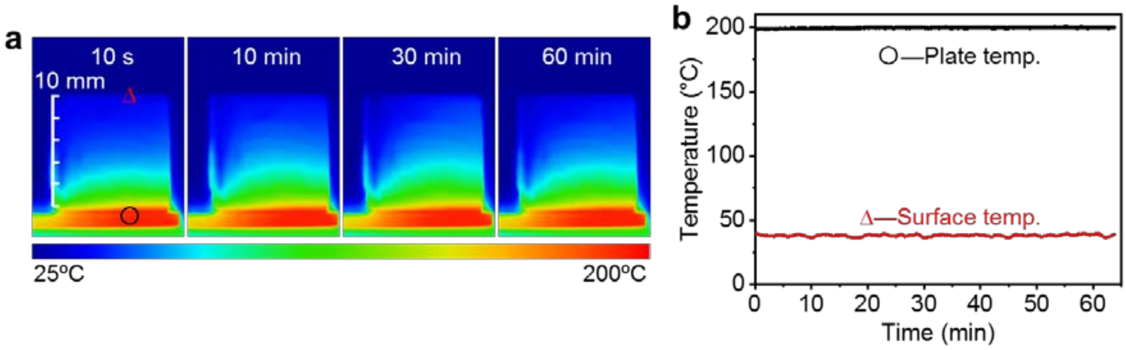

Figure S19. (a) Real time infrared images of the CMA (radial direction) placed on a hot stage of 200ºC after different time. (b) Temperature versus time curves for the surface CMA (radial direction) and the plate.

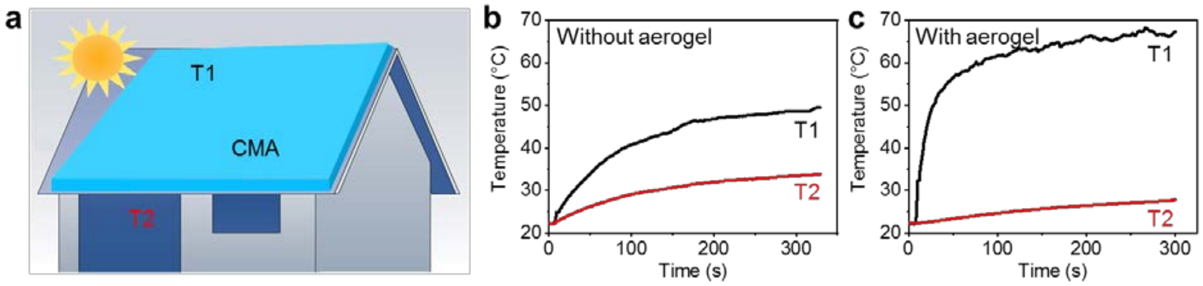

Figure S20. (a) Schematic illustration of a house model with CMA as thermal insulation material. (b, c) The internal and external temperature variations over irradiation time under sunlight for a house model with and without CMA thermal insulation materials.

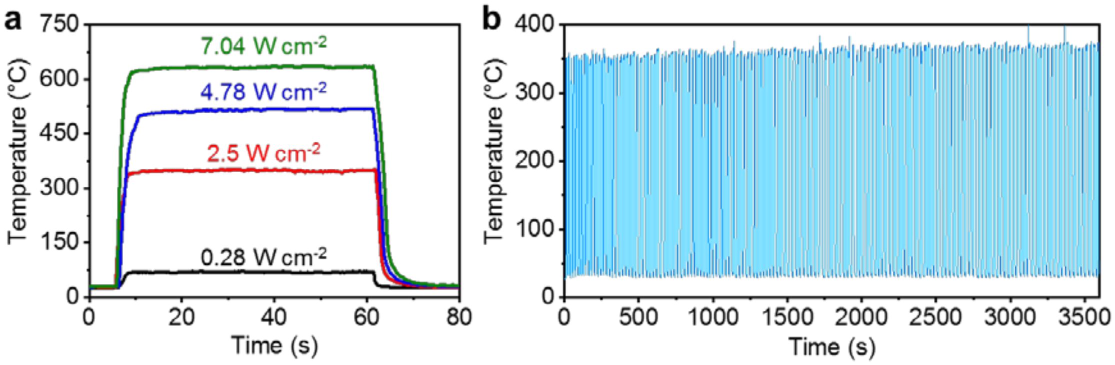

Figure S21. (a) Temperature versus time curves for the CMA that was exposed to different intensities of 808 nm near-infrared (NIR) laser. (b) Temperature versus time curves for the CMA was irradiated with 808 nm NIR laser radiation that provides an input energy of 2.5 W cm^{-2} for 200 cycles.

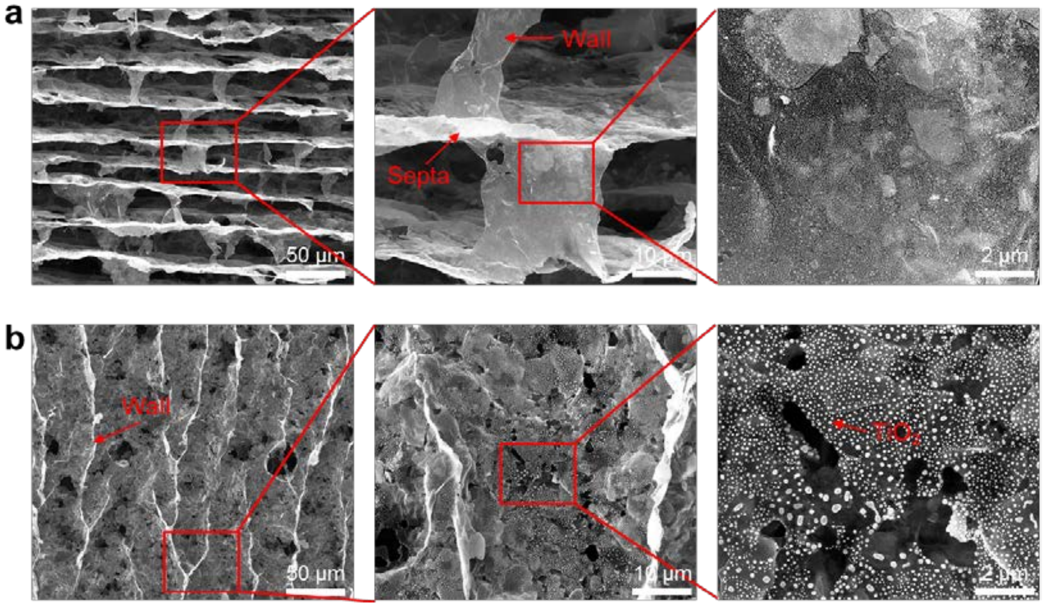

Figure S22. (a) SEM images of the cross-section of CMA after thermal shock with 1300ºC. (b) SEM images of the cross-section of CMA after thermal shock with 1300ºC.

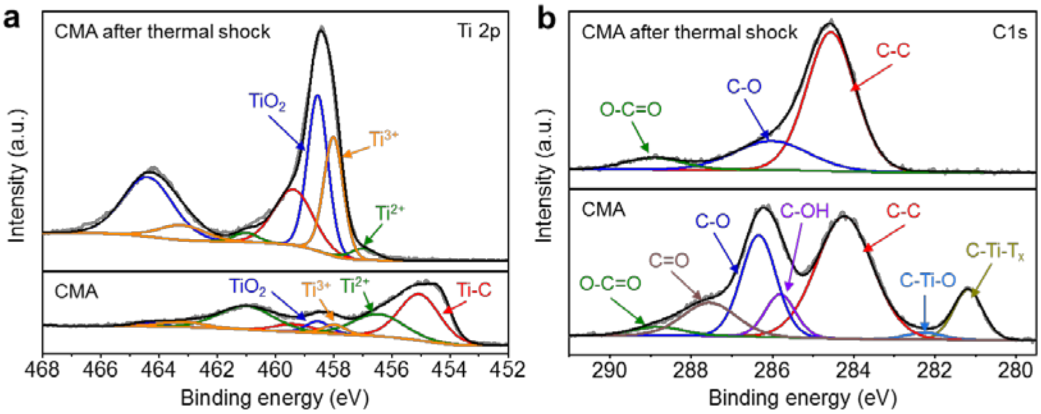

Figure S23. (a) High-resolution XPS Ti 2p spectra of CMA and CMA after thermal shock with 1300ºC. (b) High-resolution XPS C 1s spectra of CMA and CMA after thermal shock with 1300ºC.

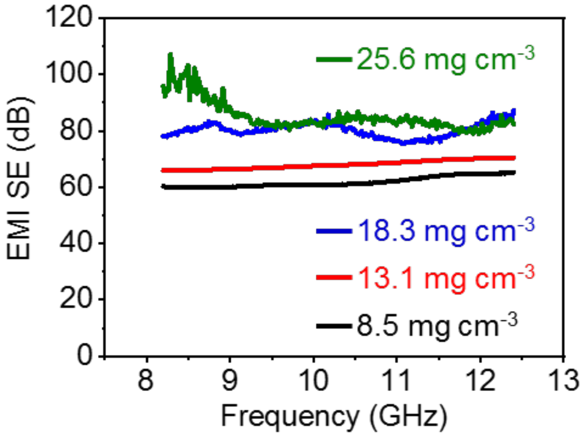

Figure S24. EMI SE values (radial direction) of the CMA with different density.

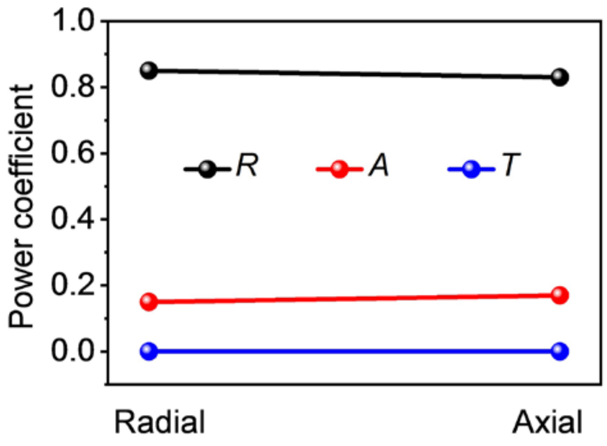

Figure S25. Power coefficients of *A*, *R*, and *T* of the CMA in both radial and axial directions.

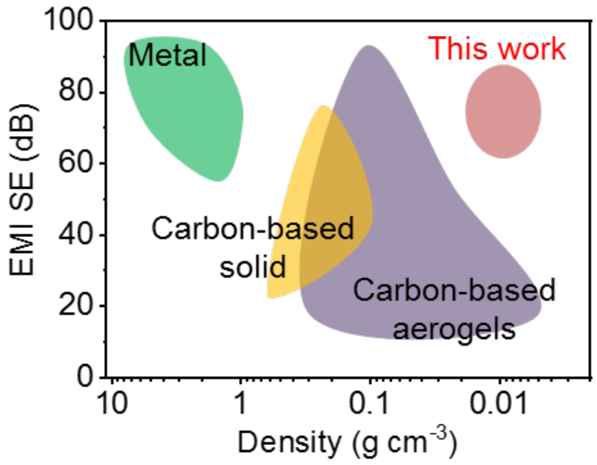

Figure S26. Comparison of EMI SE values versus density between CMA and other reported electromagnetic interference shielding materials.

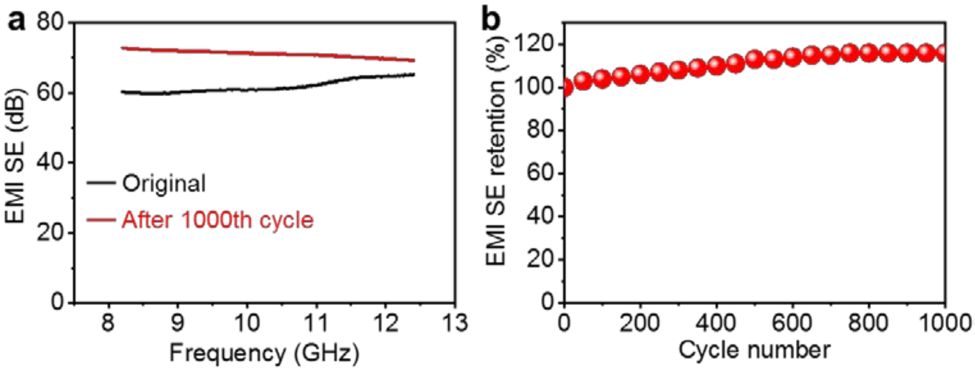

Figure S27. (a) EMI SE values of the CMA in the radial direction before and after 1000 cycles compressive at strain of 50%. (b) EMI SE retention of the CMA in the radial direction after 1000 cycles compressive at strain of 50%.

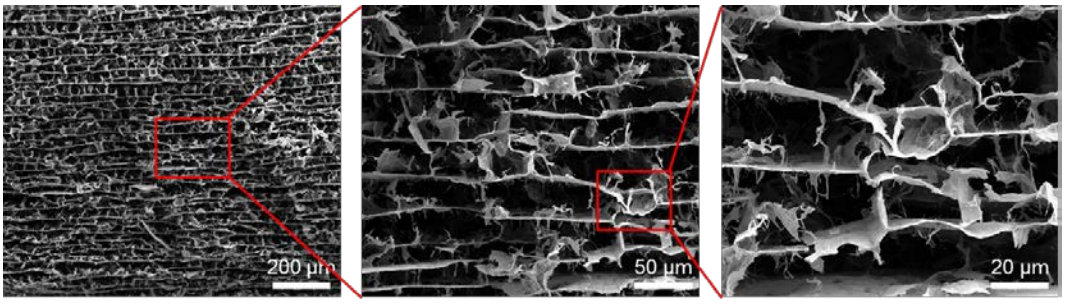

Figure S28. SEM images of the cross-section of CMA in the radial direction after 1000 cycles compressive at strain of 50%.

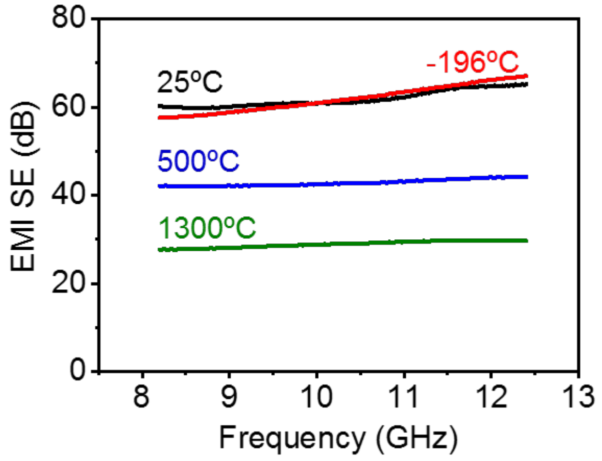

Figure S29. EMI SE values of the CMA in the radial direction after treatment in -196ºC, 25ºC, 500ºC, and 1300ºC.

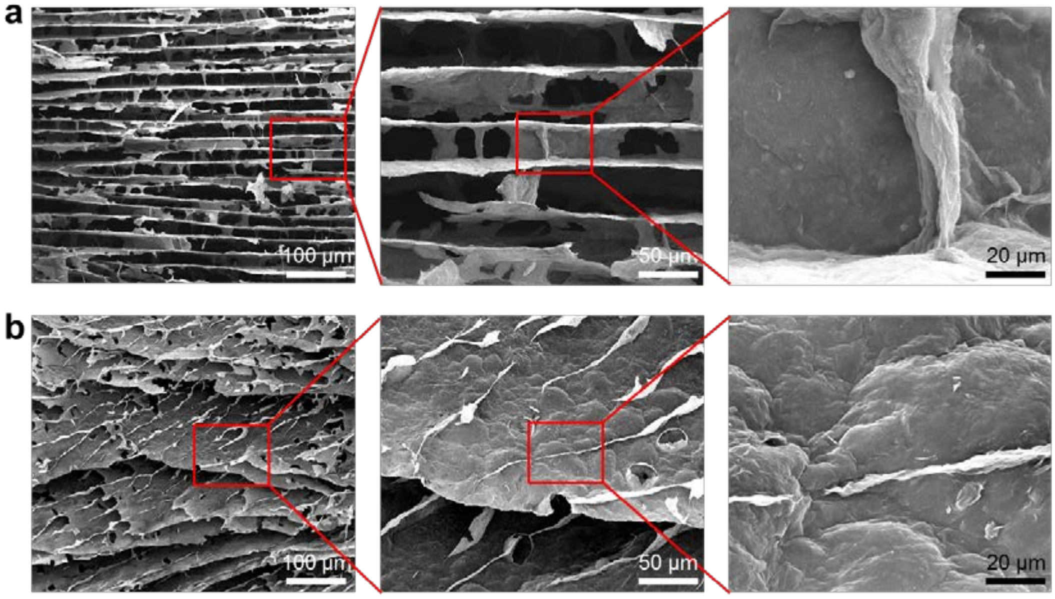

Figure S30. (a) SEM images of the cross-section of CMA after treatment in 500ºC. (b) SEM images of the top view of CMA after treatment in 500ºC.

Supplementary tables

Table S1. The atomic percentage of Ti, C, O, and F elements for CMA derived from EDS.

Sample	Ti atomic percentage (%)	C atomic percentage (%)	O atomic percentage (%)	Al atomic percentage (%)	Si atomic percentage (%)
CMA	57.9	9.9	21.1	2.4	8.7

Table S2. Comparison thermal conductivity among bioinspired and commercial thermal insulation materials.

Classification	Thermal insulation materials	Thermal conductivity (mW m^{-1} K^{-1})	Reference
	BNNS aerogel	49	22
Bioinspired	GO aerogel	56	40
	SiO_2 aerogel	41.4	41
	Wood	＞102.3	42
Commercial	Glass wool	55.1	42
	EPS foam	35	/
**This work**	**CMA**	**18.4**	**This work**

Table S3. Comparison of the compressive strength of CMA and other reported MXene aerogels in the radial and axial directions.

**Aerogel materials**	**Radial compressive strength (KPa)**	**Axial compressive strength (KPa)**	**Reference**
MXene/CNF hybrid aerogel	0.2	0.7	43
MXene/CNF/CNT aerogel	0.5	1.7	39
MXene/PINF aerogel	4.2	4.5	44
MXene/rGO aerogel	17	18	45
MXene/RGO/ANFs aerogel	22	23	46
MXene/BC aerogel	21	27	47
MXene/CNT aerogel	18	51	48
MXene/CNF aerogel	38	75	49
**CMA**	**51**	**105**	**This work**

Table S4. Comparison of the response and recovery time of CMA sensor and other MXene sensing materials.

Classification	Sensing materials	Response time (s)	Recovery time (s)	Reference
Aerogels	BC/MXene bioaerogel	0.17	0.28	47
	PINF/MXene aerogel	0.46	0.32	50
	MXene/CNC/WPU aerogel	0.15	0.32	51
	CNF/CNT/MXene aerogel	0.35	0.44	39
Hydrogels	TPU-PVAc@AgNPs/MXene nanofibrous hydrogel	0.84	0.76	52
	MXene/PU/PVA hydrogel	0.24	-	53
	MXene PpyNWs hydrogel	0.09	0.24	54
Films	MXene/BC film	0.099	0.093	55
**This work**	**CMA**	**0.2**	**0.13**	**This work**

Table S5. Thermal conductivity comparison of different aerogel materials.

Classification	Aerogel materials	Radial thermal conductivity (mW m^{-1} K^{-1})	Axial thermal conductivity (mW m^{-1} K^{-1})	Reference
Wood aerogel	Wood	29	175	42
	Polymeric wood	21	120	56
	Nanowood	32	60	57
	Wood aerogel	37	57	58
	Wood aerogel	28	115	59
	Wood-inspired aerogel	17.4	48.7	60
BNNS aerogel	hBNAGs	24	74	22
	ACA	16.9	98.3	41
GO aerogel	GO foam	41	71	40
	GO aerogel	25	37	61
	Graphene aerogels	23	65	62
	CNF-GO aerogel	15	158	63
Ceramic/SiO_2 aerogel	CSH aerogel	38	61	64
	BcF-CAs	46	82	65
	ICCAs	23	40	66
	Bioinspired cement aerogel	25	63	34
**EPS**	EPS	35	35	
**This work**	**CMA**	**17.1**	**19.7**	**This work**

Table S6. Comparison of density, EMI SE values, and SSE between CMA and other reported electromagnetic interference shielding materials.

Classification	EMI shielding materials	Density (g cm^{-3})	EMI SE (dB)	SSE (dB cm^3•g^{-1})	Reference
**Metal**	Copper	9	90	10	70
	Al foil	1.85	66	35.7	71
**Carbon based aerogel**	Graphene sponge	0.075	20	266	72
	$Ti_3C_2T_x$/RGO/ANFs	0.012	55	4583	46
	MXene/CNF aerogels	0.008	49.8	6225	43
**Carbon based sold**	rGO/PS	0.26	45	173	73
	MWCNT/PC	1.13	39	34.5	74
	GNPs/PEDOT	1.04	70	67.3	75
**This work**	**CMA**	**0.0085**	**61**	**7177**	**This work**

Movie S1. X-ray micro-computed tomography (micro-CT) image of the CMA.

Movie S2. The CMA with a thickness of 1 cm can protect the rose flower at least 1 min under the thermal shock of a butane blowtorch (1300ºC) heating.

Movie S3. The real-time high-frame-rate image of a 10-gram solid steel ball impacting CMA at high speed and rebounding.

Movie S4. Infrared image of a rose flower placed on the CMA under the thermal shock of a butane blowtorch (1300ºC).

Movie S5. A fire warning smart switchable that lights up LED when the CMA exposed to fire.
