# Supplementary material for: Isotropic thermal insulating cuttlebone-inspired MXene aerogel
Source: Natl Sci Rev. 2025 Aug 25;12(10):nwaf342. doi: 10.1093/nsr/nwaf342 (PMC12485986; doi:10.1093/nsr/nwaf342)
Supplement: nwaf342_Supplemental_Files [file nwaf342_supplemental_files.zip › Teaser text.docx]

A cuttlebone-inspired MXene aerogel with hierarchical porous structure, shows excellent thermal insulation in both radial and axial directions, rapid sensing response, robust fire resistance, and high electromagnetic interference shielding.
